# Correction: Boland, T.M.; et al. Feed Intake, Methane Emissions, Milk Production and Rumen Methanogen Populations of Grazing Dairy Cows Supplemented with Various C 18 Fatty Acid Sources. *Animals* 2020, *10*, 2380

**DOI:** 10.3390/ani11020280

**Published:** 2021-01-22

**Authors:** Tommy M. Boland, Karina M. Pierce, Jason Rowntree, Alan K. Kelly, David A. Kenny, Mary B. Lynch, Sinéad M. Waters, Stephen J. Whelan, Zoe C. McKay

**Affiliations:** 1 School of Agriculture and Food Science, University College Dublin, 4 D04 V1W8 Dublin, Ireland; karina.pierce@ucd.ie (K.M.P.); jason.rowntree@gmail.com (J.R.); alan.kelly@ucd.ie (A.K.K.); bridget.lynch@ucd.ie (M.B.L.); zoe.mckay@ucd.ie (Z.C.M.); 2 Teagasc Animal and Bioscience Department, Animal & Grassland Research and Innovation Centre, Grange, Dunsany, C15 PW93 Co. Meath, Ireland; David.kenny@teagasc.ie (D.A.K.); sinead.waters@teagasc.ie (S.M.W.); 3 Wexford Campus—IT Carlow, Summrhill, Y35 KA07 Wexford, Ireland; Stephen.Whelan@itcarlow.ie

The authors wish to make a change to the published paper [1]. In the original manuscript, the authors made a mistake when proofreading and added an incorrect Table 1. The corrected Table 1 is presented below.

The authors apologize for any inconvenience caused and the change does not affect the scientific results. The manuscript will be updated, and the original will remain online on the article webpage at https://www.mdpi.com/2076-2615/10/12/2380.

## Figures and Tables

**Table 1:** Concentrate ingredients and chemical and fatty acid composition of the concentrates and pasture offered.

Items	Pasture Herbage		Concentrate Feed ^2^		
	MP ^1^ 1	MP 2	SA	SO	LO
Ingredients (%, as fed)	-	-	-	-	-
Barley	-	-	28.5	28.5	28.5
Citrus pulp	-	-	25.0	25.0	25.0
Soy bean meal	-	-	20.4	20.4	20.4
Stearic acid	-	-	16.0	-	-
Linseed oil	-	-	-	-	16.0
Soy oil	-	-	-	16.0	-
Cane molasses	-	-	6.0	6.0	6.0
Minerals and vitamins	-	-	4.1	4.1	4.1
Chemical composition (% of DM ^3^, unless stated)					
DM (% as fed)	22.2	17.2	88.6	88.5	89.6
OM	92.3	91.4	91.9	91.5	92.7
CP	11.5	16.7	16.6	16.4	15.9
NDF	50.2	55.0	22.9	22.6	22.5
ADF	27.0	28.7	10.1	11.7	10.5
ADL	2.3	2.5	1.6	2.7	2.5
WSC (% as fed)	4.7	2.5	-	-	-
Ether extract	2.6	3.1	1.4	1.3	1.8
Gross energy (MJ/kg DM)	18.4	18.7	19.9	18.6	20.3
Fatty Acids (% of total fatty acids)					
Tetradecanoic (C_14:0_)	1.8	2.0	0.2	0.3	0.1
Hexadecanoic (C_16:0_)	19.2	18.3	6.5	12.5	7.5
c/t-9-Hexadecanoic (C_16:1_)	1.8	2.4	0	0	0
Octadecanoic (C_18:0_)	0.5	0.5	80.4	2.8	3.7
c/t-9-Octadecanoic (C_18:1_)	1.2	1.0	1.6	23.5	22.1
c-9,12-Octadecanoic (C_18:2_)	9.7	10.7	6.9	52.9	22.1
Octadecatrienoic (C_18:3_)	34.8	44.1	2.7	5.2	42.4
Others	31.0	21.0	1.7	2.8	2.1

^1^ MP = measurement period, MP 1 corresponds to 17 to 22 d post treatment introduction and MP 2 corresponds to 44 to 49 d after treatment introduction; ^2^ SA = supplementary concentrate containing 16% FW stearic acid; SO = supplementary concentrate containing 16% FW soy oil; LO = supplementary concentrate containing 16% FW linseed oil; ^3^ DM = dry matter; OM = organic matter; CP = crude protein; NDF = neutral detergent fiber; ADF = acid detergent fiber; ADL = acid detergent lignin; WSC = water soluble carbohydrates.
